# Comprehensive analysis of the expression and prognosis for IQ motif-containing GTPase-activating proteins in hepatocellular carcinoma

**DOI:** 10.1186/s12885-022-10204-3

**Published:** 2022-11-01

**Authors:** Qingqing Dai, Fei Song, Xincheng Li, Fan Huang, Hongchuan Zhao

**Affiliations:** 1 Department of Hepatopancreatobiliary Surgery and Organ Transplantation Center, Department of General Surgery, The First Affiliated Hospital of Anhui Medical University, Anhui 230032 Hefei, People’s Republic of China; 2 Department of Internal Medicine IV (Gastroenterology, Hepatology, and Infectious Diseases), Jena University Hospital, 07747 Jena, Germany; 3 Department of Urology, Jena University Hospital, 07747 Jena, Germany

**Keywords:** Bioinformatics analysis, IQGAPs, HCC, Biomarker, Prognosis

## Abstract

**Background:**

IQ motif-containing GTPase-activating proteins (IQGAPs) are a group of scaffold proteins which have been identified to be involved in tumor initiation and progression in diverse types of cancer. Clinical studies and experimental evidence suggest that IQGAPs play an essential role in hepatocellular carcinoma (HCC) progression and alterations in their expression are closely related to patient prognosis. However, the different expression patterns and prognostic values of all three IQGAP isoforms in HCC have not yet been analyzed simultaneously.

**Methods:**

We analyzed the transcriptional and survival data of IQGAPs in HCC patients using Oncomine, UALCAN, Kaplan–Meier Plotter, cBioPortal, and GeneMANIA. We further examined tumor and adjacent normal tissues from 250 HCC patients using immunohistochemistry to assess the relationship between IQGAPs expression and clinicopathological features and validate the prognostic value of IQGAPs. In addition, we analyzed transcriptional changes of IQGAPs with regards to survival data in HCC patients from the TCGA-LIHC (liver hepatocellular carcinoma) cohort to validate our results.

**Results:**

We found that the expression levels of IQGAP1 and 3 were significantly elevated in HCC tissues than in normal liver tissues, whereas the expression level of IQGAP2 was decreased in the former than in the latter. The clinical data showed that positive IQGAP1 expression was associated with larger tumor size, advanced tumor-node-metastasis (TNM) stage, poor relapse-free survival (RFS), and overall survival (OS), and positive IQGAP3 expression was associated with poorer tumor differentiation, RFS, and OS. Conversely, positive IQGAP2 expression predicted less tumor numbers and microvascular invasion, as well as higher RFS and OS in these patients.

**Conclusions:**

IQGAPs may serve as new prognostic biomarkers and potential targets for precision therapy in HCC.

**Supplementary Information:**

The online version contains supplementary material available at 10.1186/s12885-022-10204-3.

## Introduction

IQ motif-containing GTPase-activating proteins (IQGAPs) are a family of evolutionarily conserved scaffold proteins consisting of IQGAP1, 2, and 3. They are involved in numerous cellular processes, including cell proliferation, migration, division, signal transduction, and cytoskeletal dynamics [1–3]. Increasing attention has been paid to the role of IQGAPs in the initiation and progression of cancers in recent years, including liver cancer, breast cancer, gastric cancer, and colorectal cancer [4–6].

Liver cancer has emerged as a major public health threat with increasing prevalence globally. It is the sixth most diagnosed cancer and the third leading cause of cancer-related deaths worldwide [7]. Hepatocellular carcinoma (HCC) is the most prevalent type of liver cancer, accounting for more than 90% of liver cancer cases [8]. By 2025, liver cancer is anticipated to affect more than one million individuals annually [8]. Despite tremendous progress in the management and therapy of HCC, including earlier detection and alternative adjuvant therapies over the last decade, a high postoperative recurrence rate of HCC contributes to an unfavorable prognosis, with a recurrence rate as high as 70% within 5 years [9]. Current biomarkers for predicting prognosis are limited because of the tumor heterogeneity in HCC. Therefore, it is imperative to discover new biomarkers as prognostic indicators to improve prognosis, and to survey patients at a high risk of recurrence to reduce disease-related mortality.

IQGAPs are considered to play a comprehensive and distinct role in HCC and therefore warrant more research to explore the concrete mechanisms of IQGAPs in HCC [10]. IQGAP1 has been shown to be upregulated in HCC as well as being involved in the oncogenic process [11–15]. Xu et al. reported that IQGAP1 could interact with CDC42 to stimulate cell division, thus promoting the proliferation of HuH-7 cells [11]. Furthermore, IQGAP1 overexpression in HCC cells increased their invasive and migratory abilities, whereas silencing IQGAP1 decreased these abilities [16]. In contrast, IQGAP2 is thought to play the opposite role of IQGAP1 in HCC, displaying functions resembling a tumor suppressor [17–19]. Xia et al. found that IQGAP2 expression was decreased in HCC tissues and low expression was associated with an advanced tumor stages and poor prognosis [17]. Moreover, IQGAP2 deficiency in the liver predisposes mice to HCC development [20]. IQGAP3 is also considered to be oncogenic in HCC. IQGAP3 regulates DNA synthesis and mitosis in HepG2 cells and promotes cell proliferation and tumor growth in mice [21, 22].

Advances in microarray and RNA sequencing technologies have revolutionized biomedical research. In human HCC, dysregulated expression levels of IQGAPs and their relationship with clinicopathological features and prognosis have been partially reported; however, bioinformatics analysis has not been applied to explore the role of IQGAPs in HCC. Therefore, in this study, we analyzed the expression of IQGAP members in HCC based on various databases, explored their correlation with the clinicopathological characteristics and prognosis of HCC patients, and validated the results with our clinical data and specimens.

## Material and methods

### Patient enrollment

Two hundred fifty patients who underwent surgical treatment for HCC between January 2014 and December 2017 at the First Affiliated Hospital of Anhui Medical University were identified and included in this study. All patients’ tumor and adjacent normal tissues were obtained from the Department of Pathology of the First Affiliated Hospital of Anhui Medical University. Clinicopathological data, postoperative outcomes, and follow-up data were recorded for all patients. Telephone interviews and clinical records were reviewed to investigate the long-term survival of the patients, with a final follow-up date of February 2022.

### Oncomine database

Oncomine is a cancer microarray database and web-based data-mining platform that facilitates fast interpretation of a gene’s probable role in cancer and aids in genome-wide expression investigations. We used the ONCOMINE database (https://www.oncomine.org, Compendia biosciences, Ann Arbor, MI, USA) to compare the expression of IQGAPs in various cancer subtypes.

### Analysis of the Cancer genome atlas (TCGA) data using UALCAN browser

UALCAN is an exhaustive, user-friendly, and interactive web resource for OMICS data analysis [23]. UALCAN is intended to provide easy access to publicly available cancer OMICS data (TCGA, MET500, CPTAC, and CBTTC) and to enable users to uncover biomarkers or validate candidate genes of interest in silico. The expression of IQGAPs between different pathological stages and grades of HCC was analyzed using the interactive web server UALCAN (http://ualcan.path.uab.edu/index.html), according to a standard processing pipeline, and Student’s t-test or one-way analysis of variance was used to generate a *p*-value for statistical analysis.

### cBioPortal

The cBioPortal (www.cbioportal.org) is a comprehensive cancer data analysis tool [24, 25]. It provides an online analysis of various data types, including gene mutations, copy number variations, mRNA expression, and protein phosphorylation. cBioPortal was used to collect the mutation data of Liver Hepatocellular Carcinoma (TCGA, Firehose Legacy) for IQGAPs, and the mRNA expression data for IQGAPs and its frequently altered neighbor genes was collected for pathway-enrichment analysis.

### Kaplan–Meier plotter

The prognostic value of IQGAP mRNA levels in HCC was analyzed using an online database, the Kaplan–Meier plotter (www.kmplot.com) [26]. For the comparison of overall survival (OS), disease-specific survival (DSS), progression-free survival (PFS), and relapse-free survival (RFS) of HCC patients, the samples were divided into two groups based on the median expression (high versus low) and analyzed using the log-rank test. The calculated *p*-value, hazard ratio (HR) with 95% confidence intervals (CIs), and the number-at-risk cases are shown in the Kaplan–Meier survival plot.

### Biological functions analysis

KOBAS 3.0 (http://kobas.cbi.pku.edu.cn/) [27], a web-based tool for gene/protein functional annotation and pathway enrichment analysis, was used to predict the functions and pathways of the changes in IQGAPs and their frequently altered neighbor genes in HCC patients.

### String

STRING (https://string-db.org/) is a database of protein-protein interactions (PPIs) [28], which can be used to predict a comprehensive and global network for a customized protein list. In this study, the IQGAPs network analysis was performed using STRING.

### GeneMANIA

GeneMANIA (https://genemania.org/) is a resource-rich website containing gene information, analyzing gene lists, and prioritizing genes for functional assays with a high accuracy of the prediction algorithm [29]. This was used to indicate the predictive value of IQGAPs.

### Immunohistochemistry and evaluation

Tumor and normal tissue samples were obtained from paraffin-embedded specimens of tumors and adjacent normal tissues stored at the Department of Pathology of the First Affiliated Hospital of Anhui Medical University. 5-μm sections from paraffin-embedded specimens were immunohistochemically stained with Immunohistochemical kit DAB chromogenic agent (G1211, Servicebio, China). The sections were deparaffinized in BioDewax and Clear Solution, rehydrated with graded ethanol, and then placed in a repair box filled with citric acid (PH6.0) antigen retrieval buffer for antigen retrieval in a microwave oven. 3% hydrogen peroxide was applied to quench endogenous peroxidase activity, and 3% BSA was added to reduce non-specific binding. The sections were then placed flat in a wet box and incubated with primary antibodies overnight at 4 °C. Primary antibodies bought from Proteintech are as follows: IQGAP1 (1:200, 22,167–1-AP), IQGAP2 (1:50, 55,189–1-AP), and IQGAP3 (1:200, 25,930–1-AP). Then, the sections were incubated with horseradish peroxidase-conjugated secondary antibody for the corresponding species of primary antibody. DAB color developing solutions were added into sections, and the color developing time was controlled under the microscope. The sections were counterstained with hematoxylin and dehydrated with graded ethanol. At last, the sections were mounted with SweSuper Clean BioMount Medium and visualized under a microscope. Immunohistochemistry staining of IQGAP1/2/3 were evaluated by pathologists, and staining for each antibody was considered positive if more than 10% of the cells stained strongly in their cytoplasm.

### Statistical analysis

SPSS 26.0 and GraphPad Prism 8 were used for statistical analysis and graphing, respectively. The relationship between IQGAPs and clinicopathological features was analyzed using the chi-square test. Pearson’s contingency coefficient C were used for the analysis of the correlation between IQGAPs immunohistochemistry staining. Univariate and multivariate analyses of RFS and OS were performed using the Cox proportional hazards model, and the factors significantly associated with survival in the univariate analysis were further subjected to forward stepwise multivariate Cox regression analysis. RFS and OS curves were analyzed using the Kaplan–Meier method, and differences between survival curves were tested using the log-rank test. Statistical significance was set at *p* < 0.05.

## Results

### Differential expression of IQGAPs in HCC patients

**Fig. 1:**
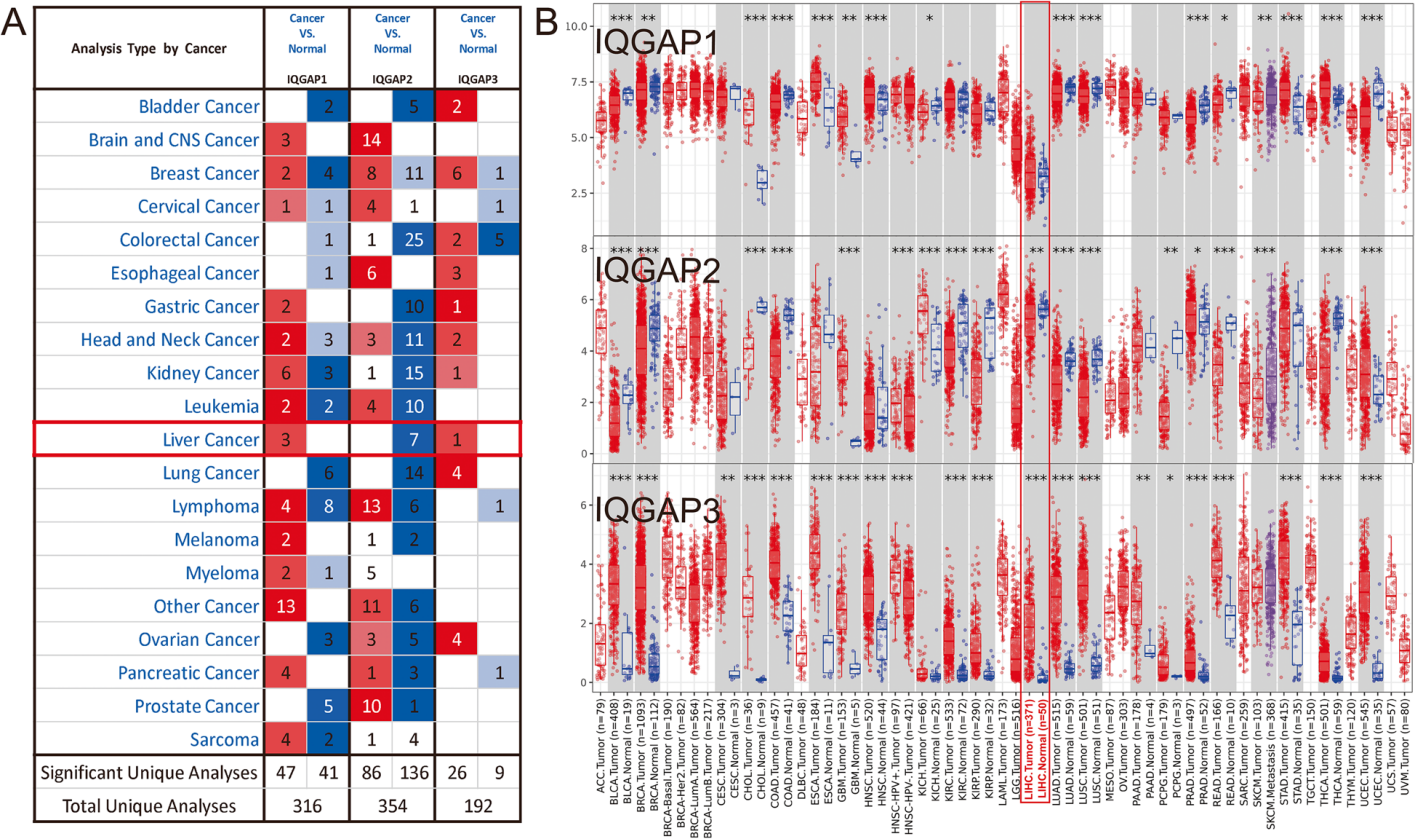
IQGAPs expression in HCC patients. **A** The transcription levels of IQGAPs in different cancer types (Oncomine). The graphic shows the numbers of datasets with statistically significant alterations in the mRNA expression of the target gene: upregulated (red) and downregulated (blue). The following criteria were used: *p*-value: 0.01, fold change: 2, gene rank: 10%, data type: mRNA, analysis type: cancer vs. normal tissue. **B** Expression of IQGAPs in the TCGA database

To investigate the expression of IQGAPs in HCC patients, the Oncomine database was used to analyze the mRNA expression levels of different IQGAPs in HCC and normal tissues. IQGAP1 and IQGAP3 expression was significantly elevated in HCC tissues, whereas IQGAP2 expression was dramatically decreased (Fig. 1A). Details of fold changes and *p* -values are presented in Supplemental Table 1. The results from the TCGA dataset were visualized (Fig. 1B). The results showed that IQGAP1 tended to be elevated in HCC tissues, but the difference was not statistically significant. IQGAP2 and IQGAP3 levels were found to be significantly decreased and increased in HCC tissues, respectively.

### Relationship between mRNA levels of IQGAPs and progression and prognosis in HCC patients

**Fig. 2:**
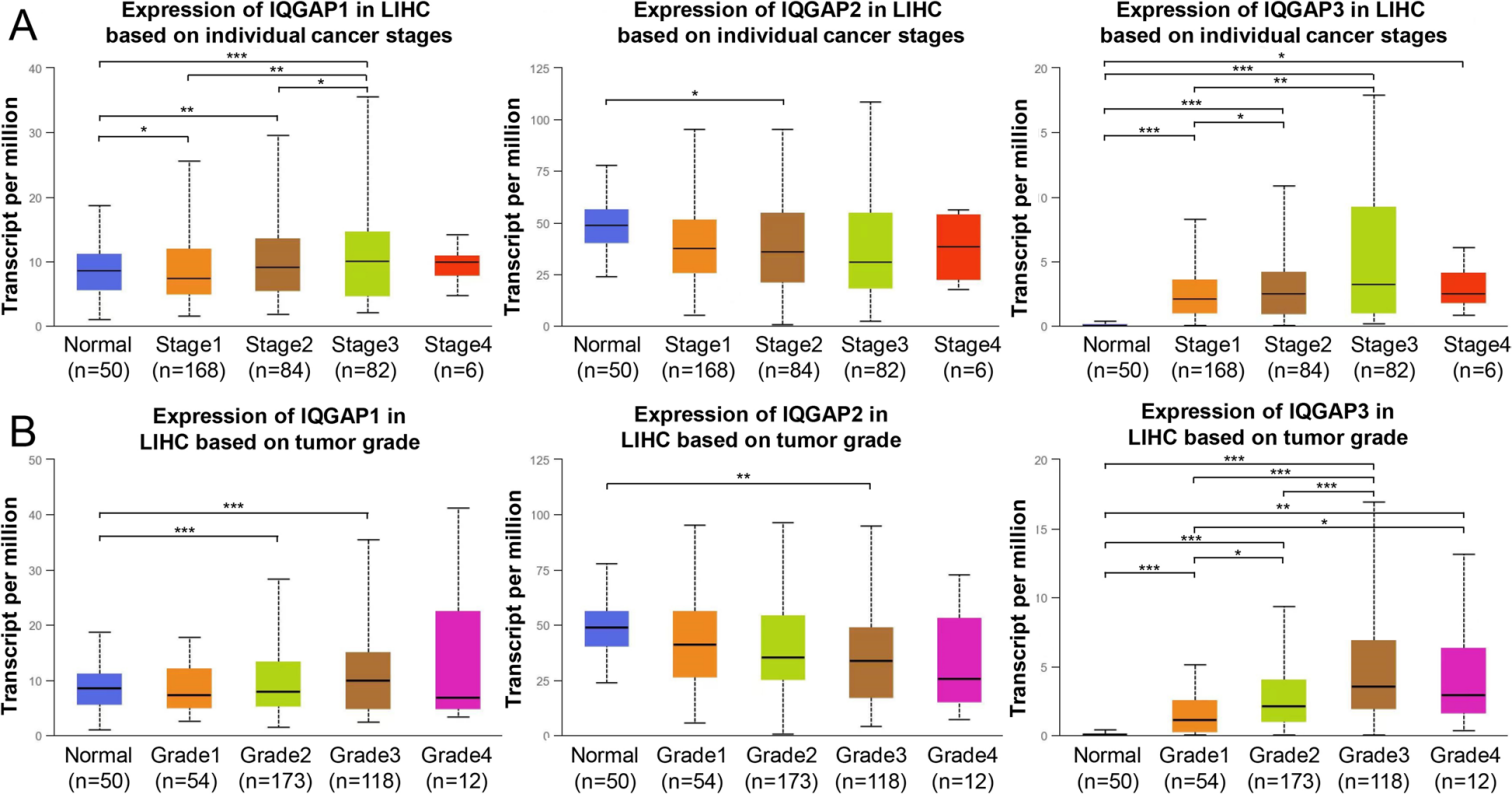
Relationship between mRNA levels of IQGAPs and progression in HCC patients. Correlations between IQGAPs expression and pathological stages (**A**) and grades (**B**) in HCC patients (UALCAN)

**Fig. 3:**
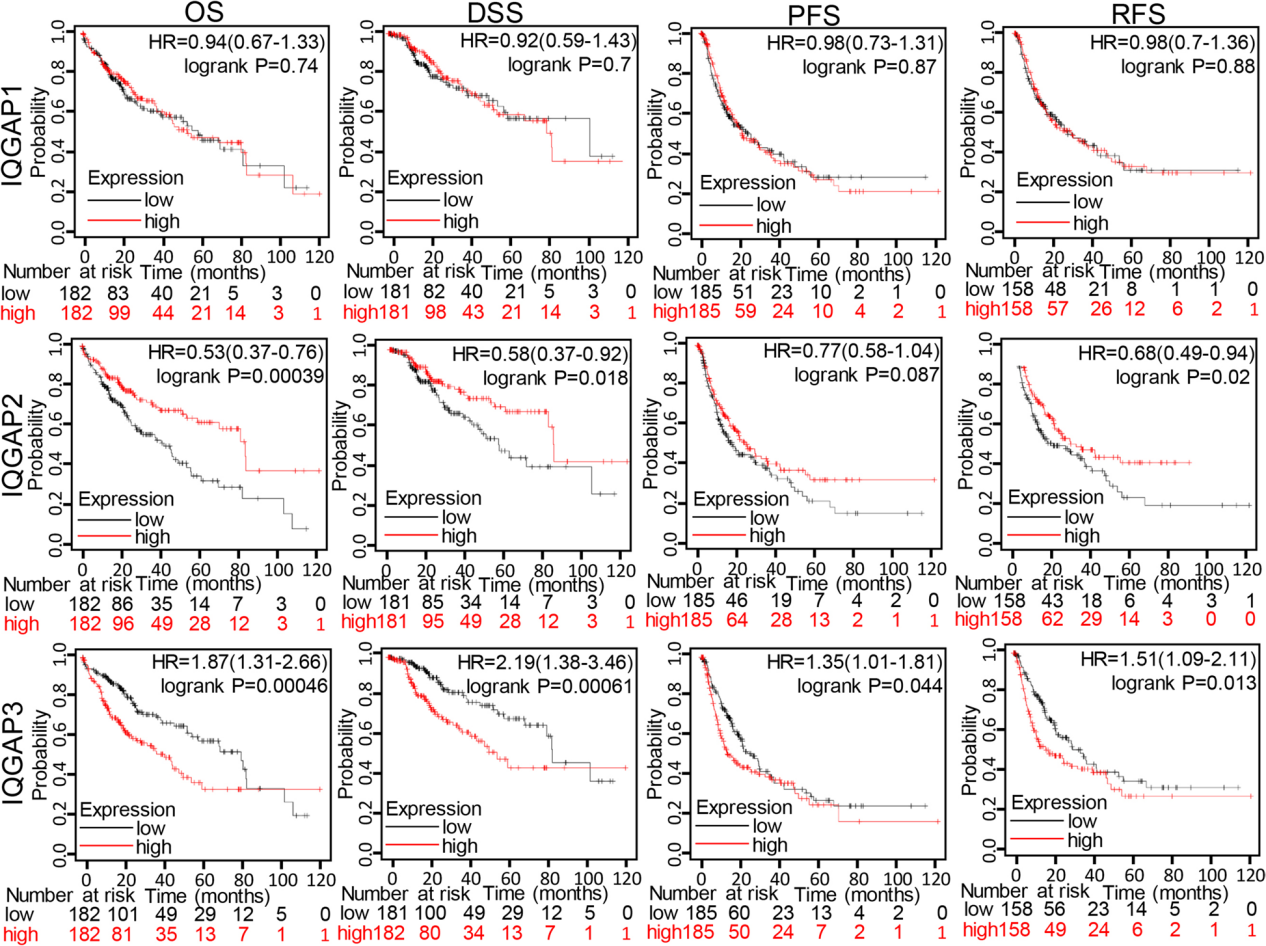
Relationship between mRNA levels of IQGAPs and prognosis in HCC patients (Kaplan–Meier plotter)

We also analyzed the relationship between IQGAP expression and tumor stage and grade of HCC in the TCGA-LIHC (liver hepatocellular carcinoma) cohort using the UALCAN. IQGAP1 was significantly increased in stage2 and 3 compared to the normal group and significantly decreased in stage1 (Fig. 2A). IQGAP2 was decreased in stage1 to 4 compared to the normal group, but only stage2 was statistically different. IQGAP3 was significantly increased in stage1 to 4 compared to the normal group. The analysis results of tumor grade were similar to those of tumor stage (Fig. 2B), IQGAP1 was significantly increased in grade2 and grade3 compared to the normal group, and IQGAP2 decreased in grades1 to 4 compared to the normal group, but only grade3 showed a statistical difference. IQGAP3 was significantly increased in stages1 to 4 compared to the normal group. We further evaluated the prognostic value of IQGAPs in HCC patients by analyzing the relationship between mRNA expression levels of IQGAP1–3 and OS, DSS, PFS, and RFS using the Kaplan–Meier Plotter tool. The results demonstrated that a higher IQGAP2 level was significantly correlated with longer OS, DSS, and RFS, while a higher IQGAP3 level was significantly correlated with shorter OS, DSS, PFS, and RFS (Fig. 3).

### Genetic alteration, co-expression, and interaction analyses of IQGAPs in HCC patients

**Fig. 4:**
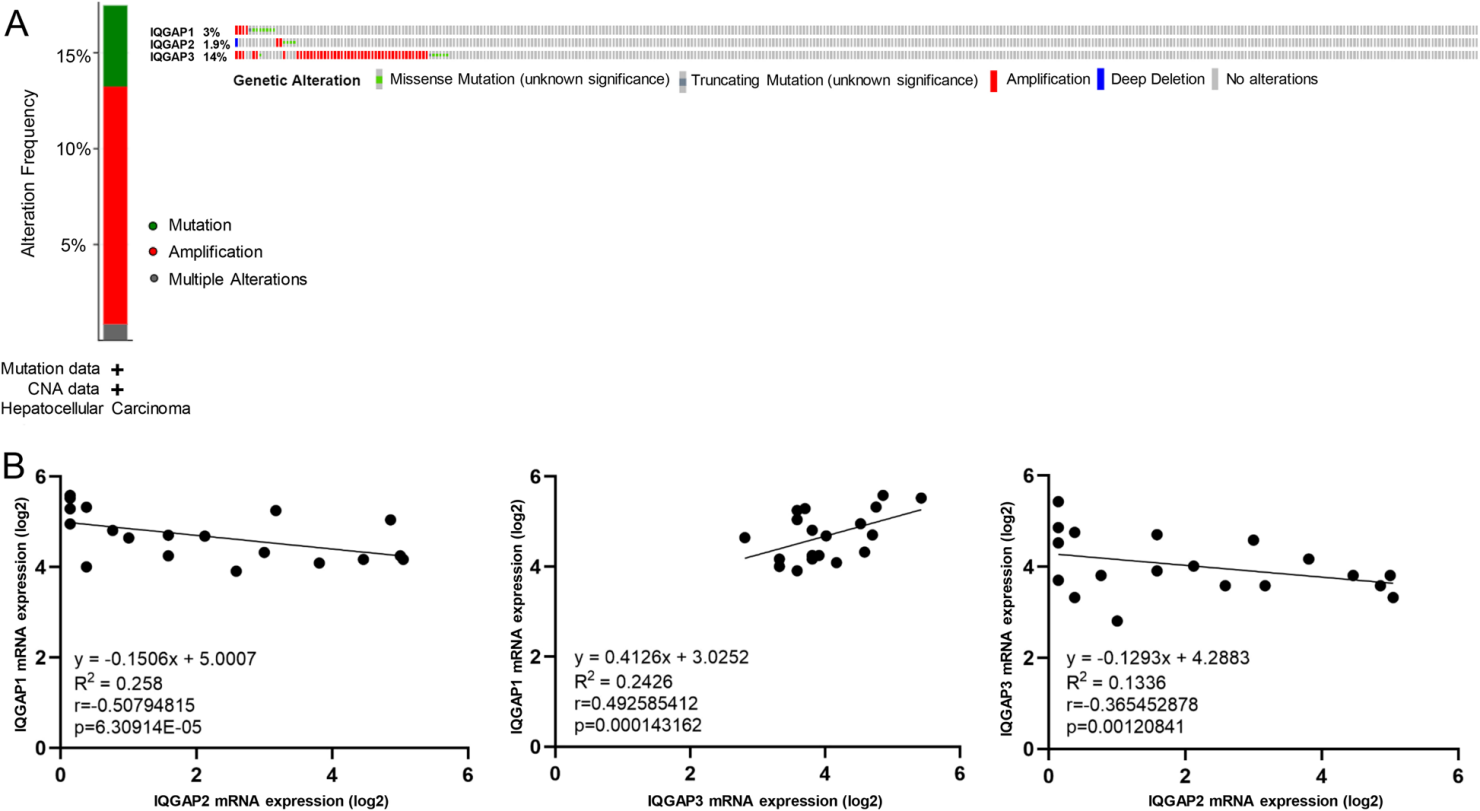
IQGAPs gene mutation and co-expression analyses in HCC. **A** Summary of alterations in different expressed IQGAPs in HCC. **B** Correlations among IQGAPs in HCC cell lines from the Cancer Cell Line Encyclopedia

**Fig. 5:**
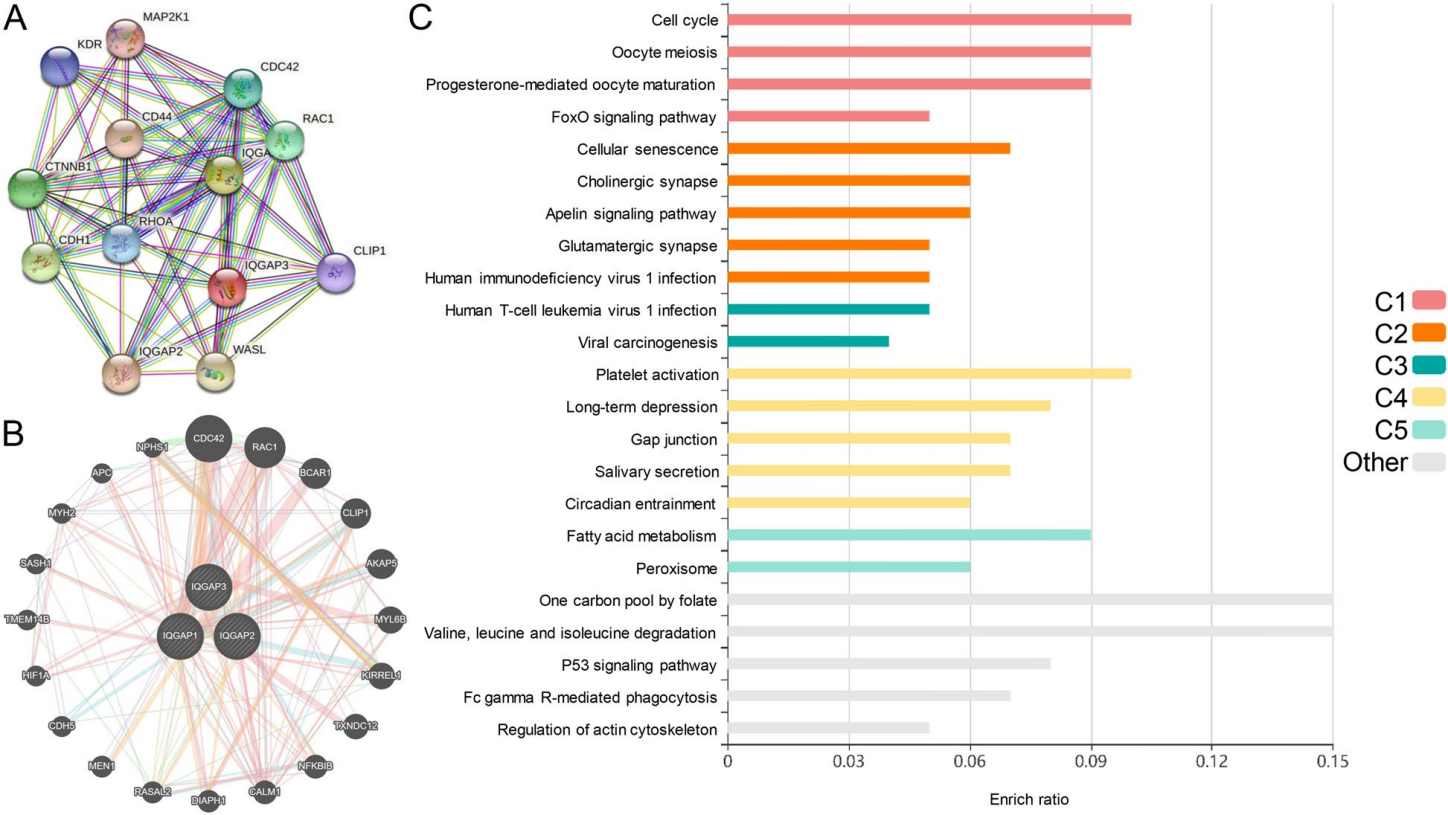
IQGAPs expression analyses in HCC (cBioPortal and STRING). **A** Protein–protein interaction network of different expressed IQGAPs. **B** The network for IQGAPs and the 20 most frequently altered neighbor genes. **C** KEGG pathway enrichment analysis of IQGAPs

Since our results suggest that aberrantly expressed IQGAPs play a vital role in HCC and progression, we next analyzed the genetic alterations of IQGAPs in HCC patients using the cBioPortal online tool. IQGAPs were altered in 69 of 366 HCC patients (18.9%), where IQGAP1, IQGAP2, and IQGAP3 were altered in 3, 1.9, and 14% of the queried HCC samples, respectively (Fig. 4A). We calculated the correlations between IQGAPs in HCC cell lines by analyzing mRNA expression (RNA Seq V2 RSEM) using the RNA-seq data of 1019 human cancer cell lines from the Cancer Cell Line Encyclopedia, incorporating Pearson’s correction. The results showed a positive correlation between IQGAP1 and IQGAP3 and a negative correlation between IQGAP2 and IQGAP1/3 (Fig. 4B) in HCC cell lines. Although tests are all statistically significant, the level of correlation is low. We then constructed a PPI network with IQGAPs at the center and 10 representative hub genes (CDC42, RAC1, CLIP1, WASL, RHOA, CDH1, CTNNB1, CD44, KDR, and MAP 2 K1) at the periphery to explore potential interactions among them (Fig. 5A). We also constructed a network of IQGAPs and the 20 most frequently altered neighboring genes (Fig. 5B). The PPI results indicated that the Rho family of GTPases (CDC42, RAC1, and RHOA) is closely associated with alterations in IQGAPs. To further understand the potential mechanisms of IQGAPs in HCC, we performed KEGG analysis of IQGAP-co-expressed genes [30, 31]. The results suggested that IQGAPs function in HCC by participating in the cell cycle, viral carcinogenesis, gap junction, and regulation of actin cytoskeleton (Fig. 5C).

### Verification of correlation between IQGAPs expression and clinicalpathological features

**Fig. 6:**
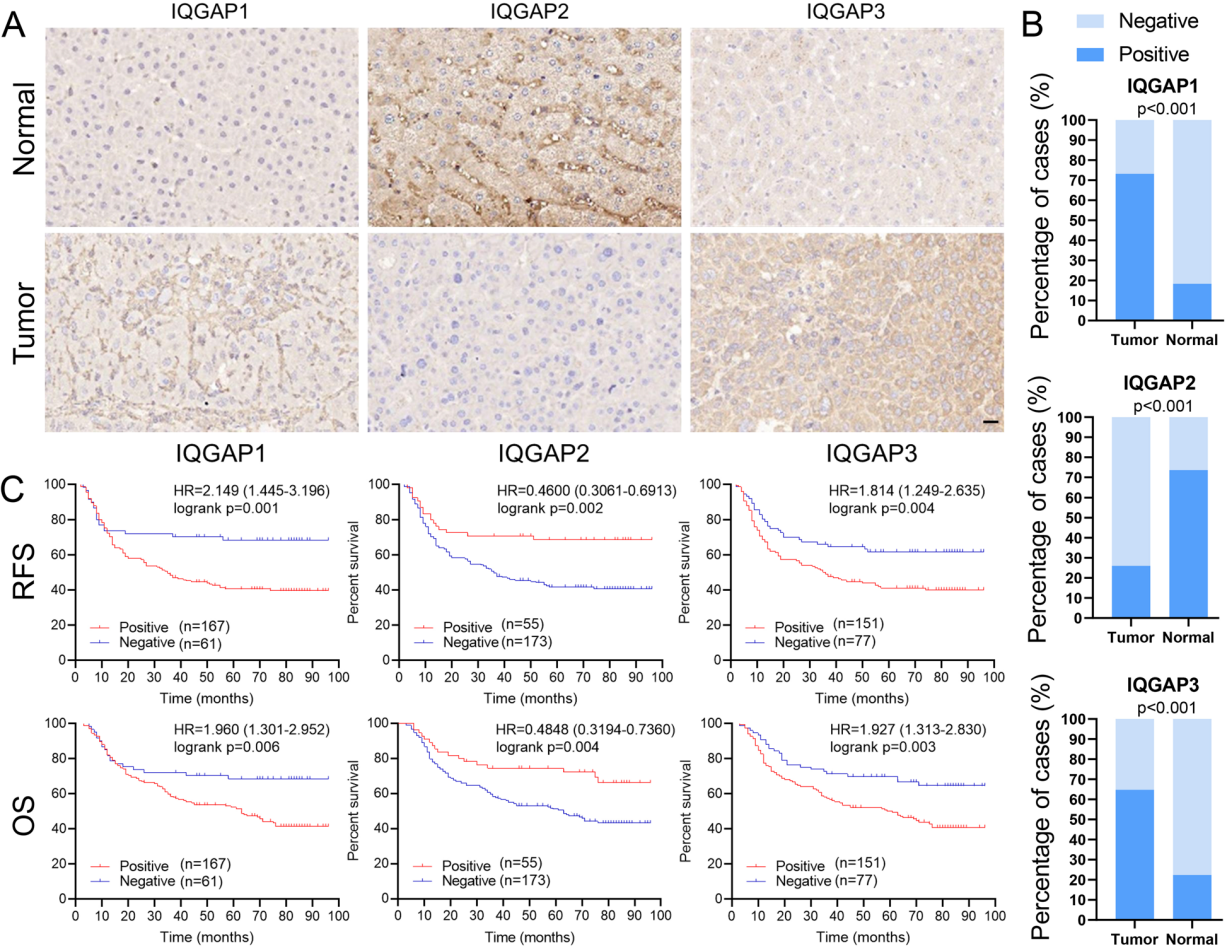
Immunohistochemistry and Kaplan–Meier relapse-free survival and overall survival analysis of respective IQGAPs expression in HCC patients. **A**, **B** Representative images and distribution frequencies of IQGAPs of tumor and normal tissues in HCC patients. Scale bar = 20 μm. **C** The relapse-free survival and overall survival of HCC patients with positive and negative expression of respective IQGAPs based on immunohistochemistry

We performed immunohistochemistry to detect the expression of IQGAPs in tumor tissues and matched adjacent normal tissues from HCC patients and analyzed the correlation between their expression and clinicopathological features. The results showed that, compared with normal tissues, IQGAP1 (73.2% vs. 18.4%) and IQGAP3 (64.8% vs. 22.4%) expressions were significantly elevated in HCC tissues, whereas IQGAP2 (26% vs. 73.6%) expression was significantly decreased (Fig. 6A and B). We also calculated the correlation between IQGAPs with Pearson’s contingency coefficient C based on the immunohistochemistry results, and the results showed no significant correlation among them (Supplemental Table 2). The clinical data showed that positive IQGAP1 expression was significantly associated with larger tumor size and an advanced tumor-node-metastasis (TNM) stage (Table 1). Negative IQGAP2 expression was significantly associated with multiple tumors and microvascular invasion. Positive IQGAP3 expression was significantly associated with poor tumor differentiation.

**Table 1:** Correlation between the clinicopathologic variables and IQGAP1/2/3 expression in HCC patients

Variables	N	IQGAP1		***P*** value	IQGAP2		***P*** value	IQGAP3		***P*** value
		Positive	Negative		Positive	Negative		Positive	Negative	
**Age**				0.332			0.073			0.561
>55	165	124	41		37	128		109	56	
≤55	85	59	26		28	57		53	32	
**Sex**				0.764			0.198			0.242
Male	219	161	58		54	165		139	80	
Female	31	22	9		11	20		23	8	
**HBV infection**				0.378			0.963			0.638
Present	189	141	48		49	140		124	65	
Absent	61	42	19		16	45		38	23	
**Liver cirrhosis**				0.793			0.435			0.643
Present	160	118	42		39	121		102	58	
Absent	90	65	25		26	64		60	30	
**AFP level**				0.864			0.951			0.276
>400 ng/mL	80	58	22		21	59		48	32	
≤ 400 ng/mL	170	125	45		44	126		114	56	
**Tumor size**				**0.031**			0.486			0.848
>5 cm	140	110	30		34	106		90	50	
≤ 5 cm	110	73	37		31	79		72	38	
**Tumor number**				0.766			**0.039**			0.599
Multiple	44	33	11		6	38		27	17	
Solitary	206	150	56		59	147		135	71	
**Microvascular invasion**				0.345			**0.011**			0.838
Present	55	43	12		7	48		35	20	
Absent	195	140	55		58	137		127	68	
**Differentiation degree**				0.613			0.352			**< 0.001**
Poor and moderate	173	125	48		42	131		126	47	
Well	77	58	19		23	54		36	41	
**TNM stage**				**0.016**			0.935			0.622
I and II	203	142	61		53	150		133	70	
III and IV	47	41	6		12	35		29	18	

### Verification of IQGAPs association with prognosis of HCC patients

Follow-up data were available for 228 postoperative HCC patients (22 patients were lost at follow-up). Univariate analysis showed that larger tumor size, multiple tumor numbers, microvascular invasion, poor tumor differentiation, advanced TNM stage, positive IQGAP1, negative IQGAP2, and positive IQGAP3 expressions were prognostic factors for RFS (Table 2). Multivariate analysis indicated that multiple tumor numbers, positive IQGAP1, negative IQGAP2, and positive IQGAP3 expressions were independent prognostic factors for RFS. Besides, univariate analysis showed that multiple tumor numbers, microvascular invasion, poor tumor differentiation, advanced TNM stage, positive IQGAP1, negative IQGAP2, and positive IQGAP3 expressions were prognostic factors for OS (Table 3). Multivariate analysis indicated that multiple tumor numbers, advanced TNM stage, positive IQGAP1, negative IQGAP2, and positive IQGAP3 expressions were independent prognostic factors for OS.

**Table 2:** Univariate and multivariate analysis of relapse-free survival in HCC patients

Variables	Univariate analysis			Multivariate analysis		
	HR	95% CI	***P*** value	HR	95% CI	***P*** value
Age	1.217	0.822–1.801	0.326			
Sex	1.58	0.800–3.118	0.188			
HBV infection	1.201	0.772–1.868	0.416			
Liver cirrhosis	1.176	0.799–1.730	0.411			
AFP level	1.202	0.818–1.765	0.348			
Tumor number	3.391	2.236–5.142	**< 0.001**	3.483	2.266–5.354	**< 0.001**
Tumor size	1.533	1.051–2.236	**0.026**			0.076
Microvascular invasion	1.766	1.180–2.642	**0.006**			0.112
Differentiation degree	1.646	1.044–2.597	**0.032**			0.650
TNM stage	2.179	1.387–3.424	**0.001**			0.074
IQGAP1	2.159	1.320–3.531	**0.002**	2.165	1.321–3.547	**0.002**
IQGAP2	0.459	0.275–0.768	**0.003**	0.538	0.320–0.906	**0.020**
IQGAP3	1.816	1.194–2.762	**0.005**	2.152	1.402–3.303	**< 0.001**

**Table 3:** Univariate and multivariate analysis of overall survival in HCC patients

Variables	Univariate analysis			Multivariate analysis		
	HR	95% CI	***P*** value	HR	95% CI	***P*** value
Age	1.214	0.813–1.809	0.344			
Sex	1.601	0.809–3.165	0.176			
HBV infection	1.120	0.718–1.747	0.619			
Liver cirrhosis	1.136	0.766–1.684	0.527			
AFP level	1.312	0.889–1.937	0.172			
Tumor number	3.417	2.219–5.262	**< 0.001**	3.098	1.957–4.902	**< 0.001**
Tumor size	1.444	0.983–2.121	0.061			
Microvascular invasion	1.799	1.193–2.713	**0.005**			0.183
Differentiation degree	1.767	1.090–2.867	**0.021**			0.701
TNM stage	2.503	1.587–3.948	**< 0.001**	1.953	1.212–3.147	**0.006**
IQGAP1	1.963	1.198–3.217	**0.007**	1.826	1.109–3.006	**0.018**
IQGAP2	0.484	0.289–0.812	**0.006**	0.545	0.322–0.922	**0.024**
IQGAP3	1.929	1.244–2.991	**0.003**	2.291	1.465–3.581	**< 0.001**

**Fig. 7:**
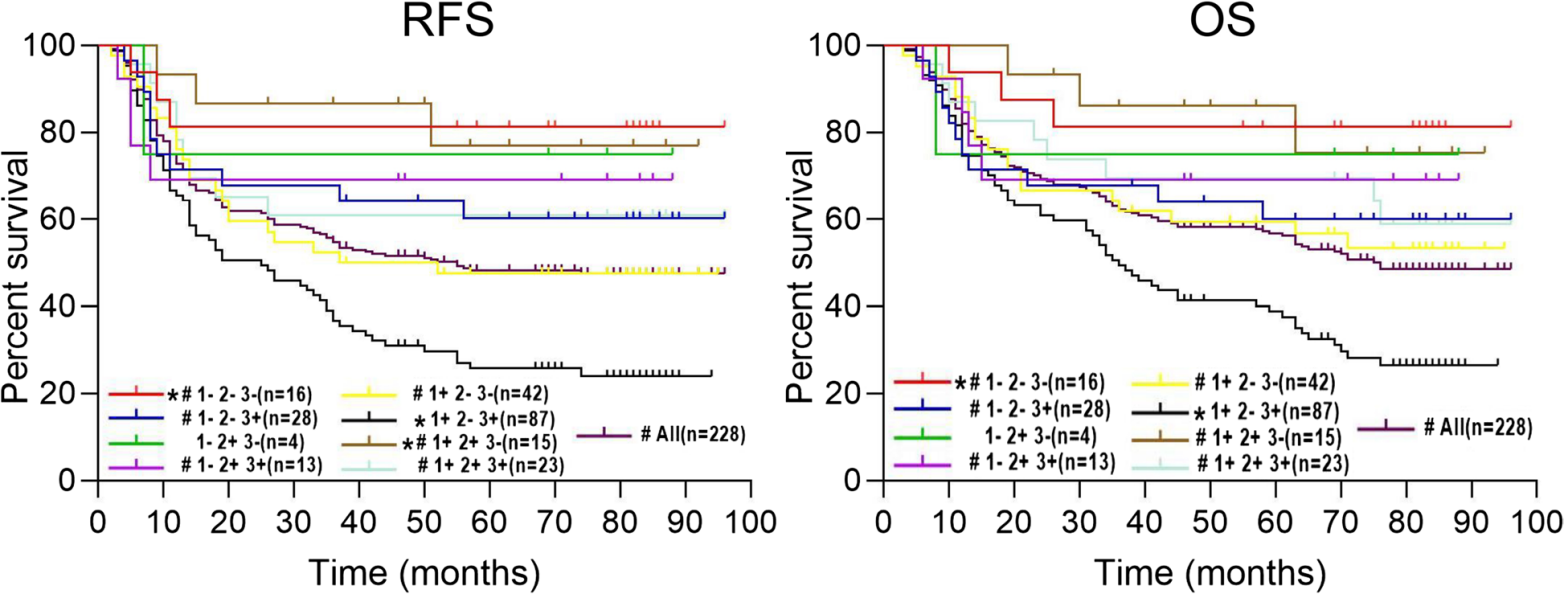
Kaplan–Meier relapse-free survival and overall survival analysis of patients with different IQGAPs expression patterns and all patients in HCC based on immunohistochemistry (1: IQGAP1, 2: IQGAP2, 3: IQGAP3, +: positive staining, −: negative staining, *p < 0.05 for the comparison with All, #*p* < 0.05 for the comparison with 1+ 2–3+)

RFS and OS curves indicated that patients with positive IQGAP1, negative IQGAP2, or positive IQGAP3 tumors based on immunohistochemistry had significantly shorter RFS and OS (Fig. 6C). Subsequently, we divided the 228 patients into eight subgroups based on IQGAP1/2/3 immunohistochemistry of tumor tissues and analyzed RFS and OS curves of nine groups including all patients and eight subgroups. The results showed that patients with IQGAP1 positive, IQGAP2 negative, and IQGAP3 positive tumors had the worst RFS and OS (Fig. 7). In addition, we analyzed the transcriptional and survival data of IQGAPs in HCC patients using the TCGA-LIHC cohort to validate our results. We divided 365 HCC patients into eight subgroups according to the mRNA median expression of IQGAPs and analyzed OS curves of nine groups including all patients and eight subgroups. The results showed that the patients in tumor tissues with high IQGAP1 expression, low IQGAP2 expression, and high IQGAP3 expression had the shortest survival time (Supplemental Fig. 1), which is consistent with our findings. Therefore, we concluded that IQGAP1/3 are oncogenes in HCC, where they promote advancement and metastasis and shorten the survival time of patients, while IQGAP2 is a tumor suppressor in HCC since its decrease in expression coincides with the clinicopathological characteristics and prognosis of patients.

## Discussion

Dysregulated IQGAPs have been reported in various cancers. The role of IQGAPs in the tumorigenesis and prognosis of HCC has been partially characterized; however, a systematic bioinformatics analysis of IQGAPs family members in HCC has not yet been performed. This study to our knowledge, is the first time to explore the expression and prognostic values of all 3 IQGAP isoforms in HCC. We hope that IQGAPs may serve as new prognostic biomarkers and potential targets for precision therapy to improve the accuracy of prognosis and prolong survival in HCC patients.

IQGAP1 has been the most researched in HCC. IQGAP1 facilitates hepatocarcinogenesis and stem cell-like properties by phosphorylating and activating FTO to upregulate the expression of transcription factors NANGO/SOX2/KLF4 in HCC [12]. Many studies and our PPI network and KEGG results suggest that the interaction between IQGAP1 and Rho family proteins plays a vital role in the initiation and progression of HCC, especially in HBV-induced HCC [3, 11, 32]. Chronic hepatitis B infection has long been considered the most prominent pathogenic factor in HCC, and the hepatitis B virus X protein (HBx) is thought to be oncogenic and promotes the progression of HCC [33, 34]. Xu et al. found that HBx enhanced proliferation and inhibited apoptosis of HCC cells and was accompanied by significantly higher expression and activity of CDC42 and IQGAP1 [11]. Later, they knocked down CDC42 using the CRISPR/Cas9 system or used the CDC42-specific inhibitor, CASIN, and found that HBx-mediated cell proliferation, anti-apoptosis, and migration were significantly reduced, coupled with a sharp decrease in IQGAP1 expression. In addition, Mo et al. found that IQGAP1 was markedly elevated in HBV-positive cells and tissues, and its expression was positively correlated with worse prognosis [32]. To verify the function of IQGAP1 in HBV-induced HCC, they overexpressed IQGAP1 in non-HBV-producing HepG2 cells and knocked down IQGAP1 in HBV-producing HepG2.2.15 cells. The results demonstrated that IQGAP1 overexpression significantly promoted anchorage-dependent growth and reduced Caspase3 levels in HepG2 cells, whereas IQGAP1 knockdown reduced the resistance of HepG2.2.15 cells to anoikis. They further found that HBV-induced ROS promoted the binding of IQGAP1 and RAC1 and subsequently activated the Src/FAK signaling pathway to promote the proliferation, migration, invasion, and anoikis resistance of HCC cells.

Clinical studies have shown that the mRNA and protein expression of IQGAP1 is significantly elevated in HCC tissues compared to normal tissues, and that elevated IQGAP1 is strongly associated with poor clinicopathological features and short postoperative survival times [35, 36]. Our immunohistochemistry results showed that IQGAP1 is localized at the site of cell-cell adhesion sites in normal liver tissues. In contrast, IQGAP1 is overexpressed in tumor tissues at the cytoplasm and cell contact sites. When overexpressed, IQGAP1 was reported to interact with β-catenin to reduce E-cadherin-mediated cell-cell adhesion and thus promote cell detachment and migration [37]. Therefore, elevated IQGAP1 in tumor tissues may contribute to poorer patient prognosis by promoting tumor cell shedding, metastasis, and invasion. Xia and Li et al. found that positive IQGAP1 expression was associated with microvascular invasion, multiple tumor lesions, large tumor size, advanced tumor stage, and poor differentiation [16, 17]. Concurrently, patients’ RFS and OS were significantly shorter. These findings are consistent with our results obtained from the Oncomine dataset, Kaplan–Meier plotter, and clinical data.

IQGAP2 is predominantly expressed in the liver, and multiple studies have shown that IQGAP2 exerts tumor-suppressive effects in a variety of cancers [5, 17, 18]. IQGAP2 inhibits epithelial-to-mesenchymal transition (EMT) and angiogenesis and promotes apoptosis in cancer cells. In addition, IQGAP2 mutant mice are more prone to spontaneous HCC formation, and more than 80% of Iqgap2^−/−^ mice develop HCC at 18–24 months of age [19, 20]. The anti-oncogenic effect of IQGAP2 may be related to its inhibition of the Wnt/β-catenin signaling pathway. β-catenin expression was inversely correlated with IQGAP2 in HCC tissues, and decreased IQGAP2 levels activated the Wnt/β-catenin signaling pathway resulting in enhanced HCC cell proliferation, migration, and EMT [19]. The expression of IQGAP2 was mostly downregulated in HCC patients. Immunohistochemistry staining showed that IQGAP2 was strongly expressed in normal tissues in the cytoplasm and cell membrane, especially in the cell-cell junctions, while it was significantly decreased in tumor tissues. It has been reported that IQGAP2 is essential for cell-cell adhesion, therefore, decreased IQGAP2 in tumor tissues also causes decreased cell-cell adhesion and promotes tumor cell metastasis and implantation to other normal liver tissues or organs [38]. Other clinical studies also showed that low expression of IQGAP2 was strongly associated with larger tumor size, multiple tumor lesions, poorer tumor differentiation, and shorter postoperative RFS and OS [17, 36]. Xia et al. found that high IQGAP1 and low IQGAP2 expression levels were independent risk factors for postoperative prognosis in HCC patients. Survival curves suggested that patients with IQGAP1^+^/IQGAP2^−^ had the worst prognosis, whereas patients with IQGAP1^−^/IQGAP2^+^ had the best prognosis [17]. These results are consistent with our findings obtained from the regression analysis and the Kaplan–Meier method, validating the distinct roles of IQGAP1 and IQGAP2 in HCC.

IQGAP3 plays a crucial role in regulating mitosis and maintaining genomic integrity and stability, and the physiological level of IQGAP3 is essential for cell proliferation and migration [39, 40]. Immunohistochemistry staining of IQGAP3 showed that it was very weakly expressed in the cytoplasm of normal liver tissues and strongly expressed in the cytoplasm of tumor tissues. Qian et al. also found that IQGAP3 expression was significantly elevated in cancerous tissues compared to that in normal tissues and was strongly associated with large tumor size, advanced tumor stage, poor differentiation, and intra- and extrahepatic metastasis in HCC [41]. In addition, serum IQGAP3 has been identified as a novel biomarker for HCC screening and diagnosis. The combination of AFP, IQGAP3, and TCP1-containing complex subunit 3 (CCT3) could increase the diagnostic accuracy of HCC by 17% compared with AFP alone [41]. Shi et al. found that IQGAP3 activates TGF-β to increase the expression of downstream proteins to enhance the migration and invasion of HCC cells [21]. Besides, IQGAP3 can also bind to protein kinase Cδ (PKCδ) to competitively inhibit the interaction between PKCδ and PKCα, allowing PKCα release, and activate PI3K/AKT signaling pathways to promote HCC cell proliferation [22]. Our results also revealed that IQGAP3 was elevated in HCC patients and was associated with shorter postoperative RFS and OS.

Despite the highly similar structure and sequence homology of IQGAPs family proteins, the results show that IQGAP1/3 and 2 play distinct roles in HCC. The relevant pieces of literature and our PPI results indicate that the Rho family may provide a putative explanation for the paradoxical phenomenon. The Rho-GTPase family is one of the critical regulators of cytoskeletal dynamics, which recycles between active form (GTP-bound) and inactive state (GDP-bound) to participate in various vital cellular functions [42]. However, IQGAPs isoforms have their GTPase binding predisposition, perhaps accounting for their different roles in hepatocellular carcinoma. For example, active CDC42 and RAC1 stimulate cell adhesion, proliferation, migration, and metastasis [43]. IQGAP1/3 appear to selectively bind to active CDC42 and RAC1, whereas IQGAP2 indistinguishably binds to both active and inactive forms [40, 44, 45]. In addition, RhoA and RhoC are carcinogenic and related to cell proliferation, migration, and invasion, whereas RhoB is a tumor suppressor and promotes cell apoptosis [46]. IQGAP1 has been reported to bind directly to active RhoA and RhoC without interacting with RhoB [47]. Therefore, the interaction between IQGAPs and Rho family deserves further investigation, which will contribute to revealing the concrete mechanism of IQGAPs in HCC and discovering new potential therapeutic targets.

## Conclusion

In this study, we systematically analyzed the expression and prognostic value of IQGAPs in HCC and validated the results using clinical data. Overall, these results suggest that the IQGAPs family plays an essential role in the progression of HCC. IQGAP1 and IQGAP3 were significantly elevated in cancer tissues and considered oncogenic factors; whereas, IQGAP2 was significantly decreased in cancer tissues and was considered a cancer suppressor. Aberrant expression of IQGAPs was significantly associated with poor clinicopathological features and prognosis. Therefore, IQGAPs may serve as a potential prognostic marker and therapeutic target for HCC; however, further investigations on the interaction between IQGAPs and Rho family are warranted to reveal the exact mechanism of IQGAPs in HCC.

## Supplementary Information

**Additional file 1: Supplemental Table 1.** The significant changes of IQGAPs expression in transcription levels between HCC and normal tissues (Oncomine Database)**. Supplemental Table 2.** Analysis of the correlation among IQGAP1/2/3 with immunohistochemistry staining. **Supplemental Fig. 1.** Kaplan–Meier overall survival analysis of IQGAPs mRNA expression in HCC patients from the TCGA datase. (1: IQGAP1, 2: IQGAP2, 3: IQGAP3, H: high expression, L: low expression, *p < 0.05 for the comparison with All, #*p* < 0.05 for the comparison with 1H 2 L 3H).

## Data Availability

All datasets generated for this study are included in Oncomine gene expression microarray database (www.oncomine.org), UALCAN (http://ualcan.path.uab.edu/index.html), cBioPortal (www.cbioportal.org), Kaplan–Meier plotter (www.kmplot.com), KOBAS 3.0 (http://kobas.cbi.pku.edu.cn/), STRING (https://string-db.org/), GeneMANIA (https://genemania.org/), and Supplementary Material. The clinical data of patients in this study are not publicly available due to limitations of ethical approval involving the patient data and anonymity but are available from the corresponding author on reasonable request.

## Abbreviations

IQGAP: IQ motif-containing GTPase-activating protein
HCC: Hepatocellular carcinoma
TCGA: The Cancer Genome Atlas
OS: Overall survival
DSS: Disease-specific survival
PFS: Progression-free survival
RFS: Relapse-free survival
HR: Hazard ratio
CIs: Confidence intervals
PPIs: Protein-protein interactions
TNM: Tumor-node-metastasis
LIHC: Liver hepatocellular carcinoma
HBx: Hepatitis B virus X protein
EMT: Epithelial-to-mesenchymal transition
CCT3: Containing complex subunit 3
PKC: Protein kinase C.
