# Supplementary material for: Comprehensive analysis of the expression and prognosis for IQ motif-containing GTPase-activating proteins in hepatocellular carcinoma
Source: BMC Cancer. 2022 Nov 1;22:1121. doi: 10.1186/s12885-022-10204-3 (PMC9628040; doi:10.1186/s12885-022-10204-3)
Supplement: Supplementary file 1 — Additional file 1: Supplemental Table 1. The significant changes of IQGAPs expression in transcription levels between HCC and normal tissues (Oncomine Database). Supplemental Table 2. Analysis of the correlation among IQGAP1/2/3 with immunohistochemistry staining. Supplemental Fig. 1. Kaplan–Meier overall survival analysis of IQGAPs mRNA expression in HCC patients from the TCGA datase. (1: IQGAP1, 2: IQGAP2, 3: IQGAP3, H: high expression, L: low expression, *p < 0.05 for the comparison with All, #p < 0.05 for the comparison with 1H 2 L 3H). [file 12885_2022_10204_MOESM1_ESM.docx]

**Supplemental Material**

**Supplemental table 1.** The significant changes of IQGAPs expression in transcription levels between HCC and normal tissues (Oncomine Database)

| **Gene** | **Type** | **Fold change** | **P value** | **t-test** | **Reference** |
| --- | --- | --- | --- | --- | --- |
| IQGAP1 | Hepatocellular Carcinoma vs. Normal | 2.312 | 9.31E-12 | 7.102 | [1] |
| IQGAP2 | Hepatocellular Carcinoma vs. Normal | -1.889 | 5.72E-05 | -5.079 | [2] |
|  | Hepatocellular Carcinoma vs. Normal | -1.562 | 1.45E-18 | -8.514 | [2] |
|  | Hepatocellular Carcinoma vs. Normal | -1.319 | 2.14E-05 | -4.691 | [3] |
|  | Hepatocellular Carcinoma vs. Normal | -1.312 | 0.001 | -3.027 | [1] |
| IQGAP3 | Hepatocellular Carcinoma vs. Normal | 1.093 | 1.27E-07 | 6.273 | [4] |
|  | Hepatocellular Carcinoma vs. Normal | 1.107 | 7.03E-20 | 9.685 | [4] |
|  | Hepatocellular Carcinoma vs. Normal | 2.385 | 8.87E-14 | 7.931 | [3] |

**Reference**

1. Mas VR, Maluf DG, Archer KJ, Yanek K, Kong X, Kulik L, et al. Genes involved in viral carcinogenesis and tumor initiation in hepatitis C virus-induced hepatocellular carcinoma. Mol Med 2009; 15(3-4):85-94. doi: 10.2119/molmed.2008.00110.

2. Roessler S, Jia HL, Budhu A, Forgues M, Ye QH, Lee JS, et al. A unique metastasis gene signature enables prediction of tumor relapse in early-stage hepatocellular carcinoma patients. Cancer Res 2010; 70(24):10202-12. doi: 10.1158/0008-5472.CAN-10-2607.

3. Chen X, Cheung ST, So S, Fan ST, Barry C, Higgins J, et al. Gene expression patterns in human liver cancers. Mol Biol Cell 2002; 13(6):1929-39. doi: 10.1091/mbc.02-02-0023.

4. Guichard C, Amaddeo G, Imbeaud S, Ladeiro Y, Pelletier L, Maad IB, et al. Integrated analysis of somatic mutations and focal copy-number changes identifies key genes and pathways in hepatocellular carcinoma. Nat Genet 2012; 44(6):694-8. doi: 10.1038/ng.2256.

**Supplemental table 2.** Analysis of the correlation among IQGAP1/2/3 with immunohistochemistry staining.

| IQGAP2  IQGAP1 | + | - |  | IQGAP3  IQGAP1 | + | - |  | IQGAP3  IQGAP2 | + | - |  |
| --- | --- | --- | --- | --- | --- | --- | --- | --- | --- | --- | --- |
| + | 45 | 138 |  | + | 117 | 66 |  | + | 41 | 24 |  |
| - | 20 | 47 |  | - | 45 | 22 |  | - | 121 | 64 |  |
| χ^2^ | 0.705 | |  | χ^2^ | 0.224 | |  | χ^2^ | 0.114 | |  |
| C | 0.053 | |  | C | 0.030 | |  | C | 0.021 | |  |
| P value | 0.401 | |  | P value | 0.636 | |  | P value | 0.735 | |  |
| +: positive, -: negative, χ2: Pearson’s chi-squared test, C – Pearson’s contingency coefficient C | | | | | | | | | | | |


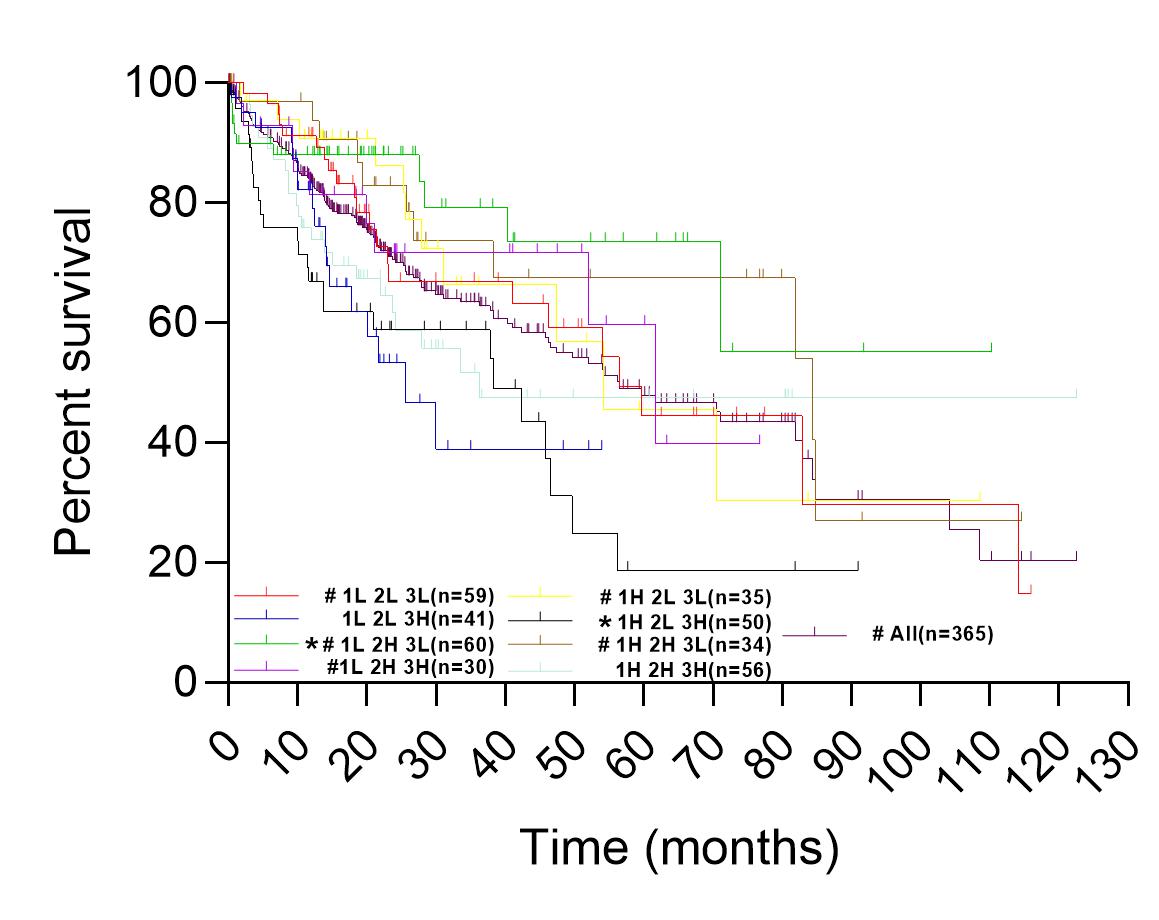


**Supplemental figure 1.** Kaplan–Meier overall survival analysis of IQGAPs mRNA expression in HCC patients from the TCGA datase. (1: IQGAP1, 2: IQGAP2, 3: IQGAP3, H: high expression, L: low expression, *p<0.05 for the comparison with All, #p<0.05 for the comparison with 1H 2L 3H).
